# Spontaneous Alpha Power Lateralization Predicts Detection Performance in an Un-Cued Signal Detection Task

**DOI:** 10.1371/journal.pone.0160347

**Published:** 2016-08-09

**Authors:** Gonzalo Boncompte, Mario Villena-González, Diego Cosmelli, Vladimir López

**Affiliations:** Escuela de Psicología, Pontificia Universidad Católica de Chile, Santiago, Chile; University of Manchester, UNITED KINGDOM

## Abstract

Focusing one’s attention by external guiding stimuli towards a specific area of the visual field produces systematical neural signatures. One of the most robust is the change in topological distribution of oscillatory alpha band activity across parieto-occipital cortices. In particular, decreases in alpha activity over contralateral and/or increases over ipsilateral scalp sites, respect to the side of the visual field where attention was focused. This evidence comes mainly from experiments where an explicit cue informs subjects where to focus their attention, thus facilitating detection of an upcoming target stimulus. However, recent theoretical models of attention have highlighted a stochastic or non-deterministic component related to visuospatial attentional allocation. In an attempt to evidence this component, here we analyzed alpha activity in a signal detection paradigm in the lack of informative cues; in the absence of preceding information about the location (and time) of appearance of target stimuli. We believe that the unpredictability of this situation could be beneficial for unveiling this component. Interestingly, although total alpha power did not differ between Seen and Unseen conditions, we found a significant lateralization of alpha activity over parieto-occipital electrodes, which predicted behavioral performance. This effect had a smaller magnitude compared to paradigms in which attention is externally guided (cued). However we believe that further characterization of this spontaneous component of attention is of great importance in the study of visuospatial attentional dynamics. These results support the presence of a spontaneous component of visuospatial attentional allocation and they advance pre-stimulus alpha-band lateralization as one of its neural signatures.

## Introduction

Oscillatory brain activity is one of the most robust macroscopic neural signatures reflecting mental processes [1]. The most prominent oscillation in human electroencephalography (EEG) is the alpha band, approximately defined between the frequencies of 8Hz and 12Hz. The cognitive role of this type of oscillatory activity has been extensively linked to attention [2]. Specifically, alpha is proposed to reflect mechanisms of attention inhibition deployed to suppress the processing of task-irrelevant or distracting stimuli [3]. Decreases in the activity of this band facilitate the visual processing of task-relevant stimuli whereas increases seem to actively inhibit processing of task-irrelevant elements, allowing an enhanced processing of relevant information. An important body of research supporting this role of attentional inhibition comes from experiments studying visuo-spatial attention orienting. Experimental strategies similar to Posner’s classical cueing paradigm [4] are normally used: a first stimulus cues about the spatial location where a subsequent target will appear. This makes subjects covertly allocate attention towards a specific side of their visual field, as shown by improved behavioral performance. This has allowed the study of the neural signatures associated with this attention process. Decreases in the amplitude of alpha over the cortical areas contralateral to the locus of attentional allocation have been reported [5,6]. Increases in alpha activity have been observed over brain regions ipsilateral to unattended spatial locations have also been reported [7–9]. Thus, alpha lateralization in response to visuospatial cuing appears as a robust signature of guided covert attentional allocation.

Homologous effects have been found by experiments using different sensory modalities for cues and stimulus also supporting a broader role of attentional inhibition for alpha band [10]. This lateralization has been reported also in purely auditory cue-stimulus tasks [11,12]. Also alpha activity related to allocation of auditory and visual attention has been shown to interact with each other [13], which has brought attention to the topic of supramodal versus modality specific attentional processes [14]. Additionally, not only alpha power has been linked to attention. The ongoing phase of alpha at the time of appearance of a stimulus has been shown to strongly correlate with whether target stimulus are detected or not [15,16].

All of the evidence above is grounded in the fact that the cue stimulus informs subjects of the location where a target stimulus will appear, thus subject’s attention is deployed accordingly to an external guide, i.e. in a deterministic way. However, recent theories about attention have emphasized that where and/or how much attention is allocated in a particular time has a relevant stochastic or non-deterministic component [17–19]. Here we wanted to test whether this probabilistic component of attention could be observed as differences in the proposed neural signatures of visual spatial attentional deployment, namely alpha band oscillation. To do this we carried out a signal detection experiment importantly lacking any cuing stimulus. Targets appeared lateralized, either left or right, with equal probability in each trial. We analyzed alpha-power across parieto-occipital areas in the time period preceding target presentation and found compelling evidence of this non-deterministic component of attention. This is shown by significant and spontaneous alpha power lateralization predicting behavioral performance.

## Methods

### Participants

Twenty-four subjects (11 females) participated in the experiment, averaging 23 years of age (SEM = 0.69), with normal or corrected to normal vision. All of them provided written consent to participate, in accordance with Pontificia Universidad Católica de Chile’s and Helsinsky’s declaration standards. This experiment was approved by the ethics committee of the psychology school of this university and was done accordingly. Data from 3 participants was discarded because of high false positive rates, indicating that they did not discriminate targets from distractors (see below). The experiment lasted for approximately 45 minutes, depending on the duration of the self-administrated rests.

### Visual Stimulation

**Fig 1:**
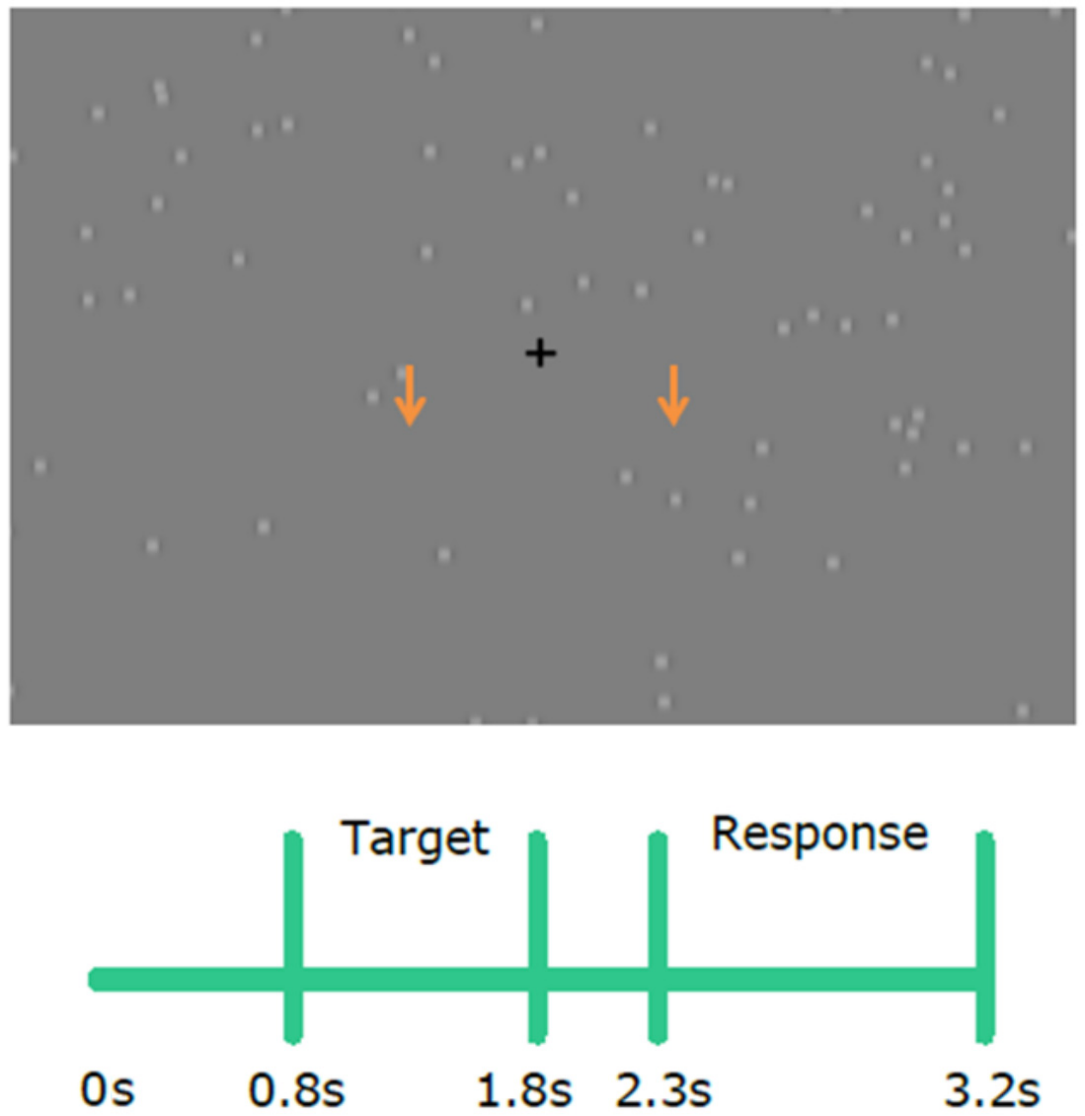
Stimulation paradigm. **(Top)** Depiction of the general scene of the visual stimulation. It shows the central fixation cross and distractors (light gray circles), each of which randomly changed location in every refresh of the screen. Arrows (not presented during the task) approximately illustrate the two areas where targets could occur. **(Bottom)** Time-line of the trial. Only during the Target period a target could occur. Response period was defined by the absence of the fixation cross, which was present during all the rest of the trial. This cued subjects to respond whether they had saw a target or not.

Stimulation was presented on a computer screen with a refresh rate of 60Hz at 60cm in front of subjects. The general scene (Fig 1) was composed of a gray uniform background and distractor stimuli. These distractors were small gray disks (0.3°) with blurred edges. The position of each distractor was chosen at random in each refresh of the screen. Each screen location had equal probability. This resulted in all distractors appearing and disappearing across the screen continuously during the trial. Targets were the same image as distractors, they were visually identical (shape, color, size, etc.) in all but one feature; they persisted on the screen consistently, moving downwards (constant speed of 11.1°/s) for 100ms (6 screen refreshes), which contrasted with the flickering distractors. Targets appeared randomly either at the left or right of the fixation cross with equal probability (6.25° of eccentricity). In this way target and distractors were almost equivalent, with movement as the distinguishing feature of targets.

The trial lasted for 3.2s and was divided into two main parts, the target period and the response period. During the target period (1s of duration, starting at t = 800ms, see Fig 1) the fixation cross was present, indicating that a target could appear in the screen. They could appear at any time during the target period, but only once per trial. After the target period, a 500ms period without targets was given. After this, the response period (900ms), indicated by the disappearance of the fixation cross, signaled subjects that they could respond to whether they had saw a target in the preceding target period or not. Subjects responded with the pressing of either the left or right hand button, indicating that they had or had not saw a target. This was counterbalanced across subjects. Importantly, they were instructed to prioritize accuracy and certainty over speed in their responses.

Only on 85% of the total amount of trials a target was presented (valid trials). On the remaining 15% (invalid trials) no target was presented and subjects were supposed to give the corresponding response. Invalid trials were introduced to assess the false positive rate (no target presented but subject report seeing one). If any subject showed more than 2.5% of false positive trials, their data were discarded from further analysis. This is because we could not be sufficiently certain of their ability to discriminate between targets and distractors. Three subjects fell under this category. Subsequent analysis and results correspond exclusively to those of valid trials. Every 10 blocks (80 trials) subjects were given a resting period without visual stimulation. The total number of trials completed by subjects with appropriate false positive rates ranged between 600 and 900: 4 subjects completed 600 trials, 16 subjects finished 800 and one did 900 trials. There was no difference between the total alpha lateralization or false positive ratings amongst these groups. This was evidenced by the individual z-scores values for these parameters for subjects who did 600 or 900 trials: none of these z-scores fell outside the 90% probability or their corresponding total distributions (every z-score was smaller than 1.65). After artifact rejection, the total mean number of usable trials per condition was 206 and 212 for Seen and Unseen conditions. Despite the different number of total trials completed, after artifact rejection, the number of usable trials for every subject was well above what has been previously shown to allow for alpha lateralization observations. [7,20,21].

### Calibration phase

**Fig 2:**
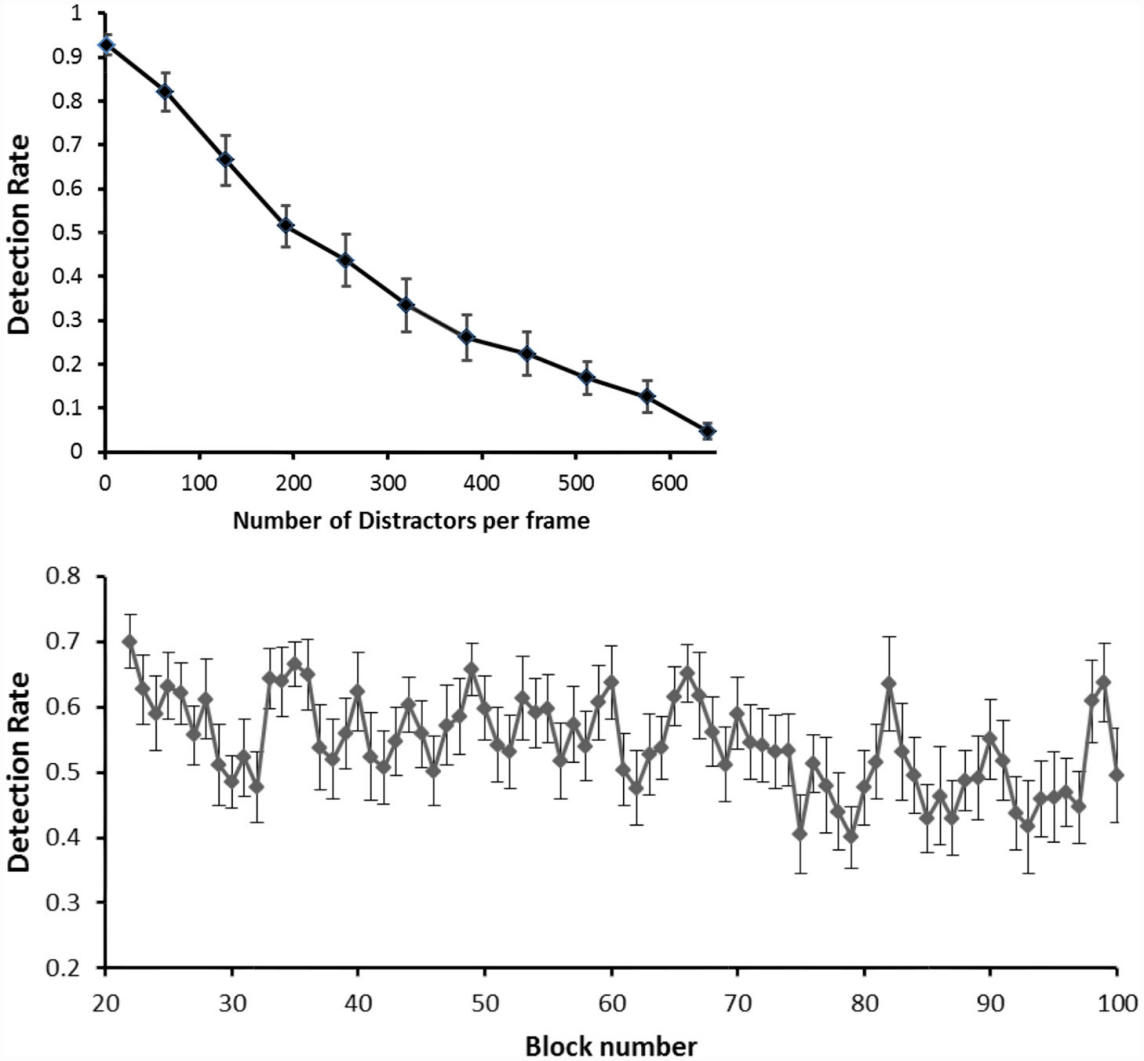
Behavioral results. (**Top**) Behavioral results during the calibration phase (first 21 blocks). It shows the strong relationship between the number of distractors *per frame* and the probability of subjects detecting the presented target. (**Bottom**) Plot illustrating the mean detection rate after the calibration phase across subjects for each block (8 trials). Error bars represent SEM.

During pilot experiments we determined that the detection rate strongly depended on the amount of distracters present per frame (Fig 2, top). To obtain a constant and balanced detection rate (similar number of Seen and Unseen trials) across trials and subjects we implemented a calibration phase, which comprised the first 168 trials of the experiment (21 blocks of 8 trials each). During this calibration phase we monotonically increased the number of distracters from 2 in block 1 to 640 in block 11 and then decreased it back from 640 in block 11 to 2 in block 21. Using the resulting information, the relation between detection rate for each subject and the number of distractors presented, we analyzed the theoretical number of distracters needed to produce a detection rate of ≈50%. Accordingly, after the calibration phase, the custom-made stimulation program fitted these results to a logistic equation, *y = 1 / (1 + e^((x-b)/32)*, where *y* is the detection rate, *x* is the number of distractors presented and *b* is the inflection point of the logistic curve, i.e., the theoretical amount of distracters necessary to yield a 50% detection rate. For each subject we used this number of distractors (*b*) for the rest of the experiment.

### EEG Recording

Electrical brain activity was measured using a digital 32-electrode EEG system (Biosemi ActiveTwo, 2048Hz sampling frequency, 24 bits) complemented with 6 additional electrodes. These were 2 mastoid reference electrodes and 4 electrooculogram (EOG) electrodes placed below and above the right eye and on the outer canthi of each eye. Vertical and Horizontal EOGs were obtained by means of bipolar derivations of the corresponding electrodes for trial rejection due to blinking. Scalp electrodes were offline referenced to the average of the two mastoid electrodes.

### Induced Alpha Power and Lateralization Analysis

Analyses were made using custom made scripts, MATLAB (The MathWorks, Inc. USA) toolboxes EEGLAB (Delorme and Makeig, 2004) and ERPLAB (Lopez-Calderon and Luck, 2014). Data was segmented into epochs from -1000ms to 1000ms relative to the beginning of the target stimulus. We used a moving window peak-to-peak threshold criterion (threshold = 200uV, window size = 200ms, step = 100ms) to automatically reject artifact-containing epochs. Raw continuous EEG data was filtered between 0.1Hz and 100Hz (Butterworth filter, 4th order). Afterwards pre-processed data was transformed into the frequency domain by means of Fourier Transform in sequential and overlapping windows (250ms) in steps of 25ms. Spectral amplitude was then computed for every time window and frequency bin (between 4Hz to 60Hz). We then normalized the resulting signal by converting it to a Z-score relative to a baseline a time windows (-870ms to -500ms relative to the onset of the target stimulus). We obtained a Z-score value for each trial, frequency bin and time window. To obtain the induced spectral power we subtracted to these Z-score values the corresponding evoked spectral power. The evoked spectral power for each subject was computed by first averaging preprocessed data, still in the time domain, and then transforming it to the frequency domain, again with Fourier Transform. The result of this subtraction is the induced spectral power [22]. This was done to analyze variations in alpha power occurring primarily in the time closely preceding (≈200ms) the appearance of the target stimulus. This is of particular relevance in the interpretation of our results. Alpha dynamics extended over longer periods of time, although of great interest, are out of the scope of the present article

**Fig 3:**
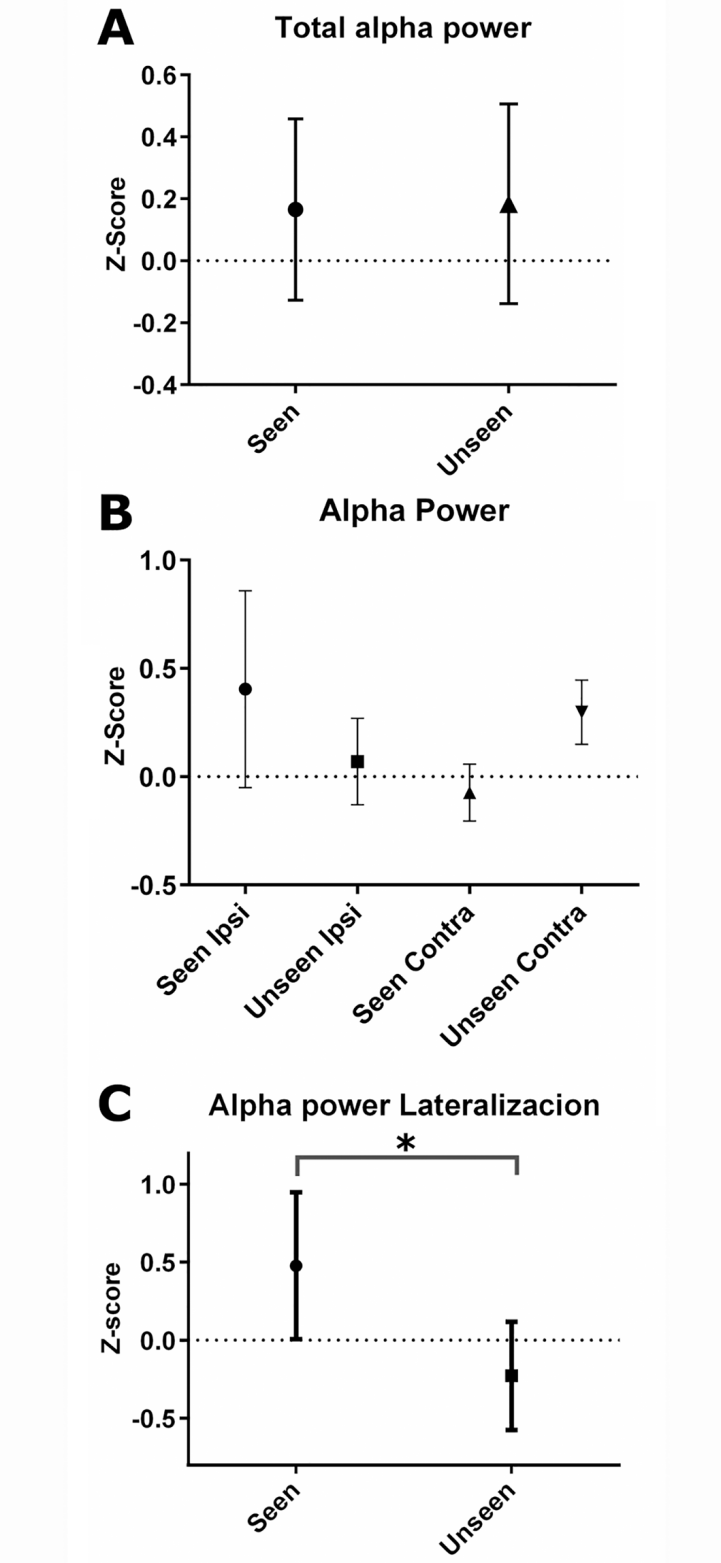
Alpha power quantification and comparison across conditions. **(A)** Shows the total parieto-occipital alpha power for Seen and Unseen conditions. **(B)** Shows the alpha power obtained accordingly to the side of presentation of the target for Seen and Unseen targets. Ipsilateral (Ipsi) corresponds to the scalp alpha power over the same side as to where the target was presented. Contralateral (Contra) corresponds to the alpha power of the opposite side to where the target was presented. **(C)** Depicts the lateralization effect for Seen and Unseen targets. Lateralization was calculated as the difference between Ipsilateral and Contralateral alpha power for both conditions. The significant statistical different among groups, as tested by Wilcoxon matched-pairs signed rank test, is shown with an asterisk. All error bars represent the 95% confidence interval.

**Fig 4:**
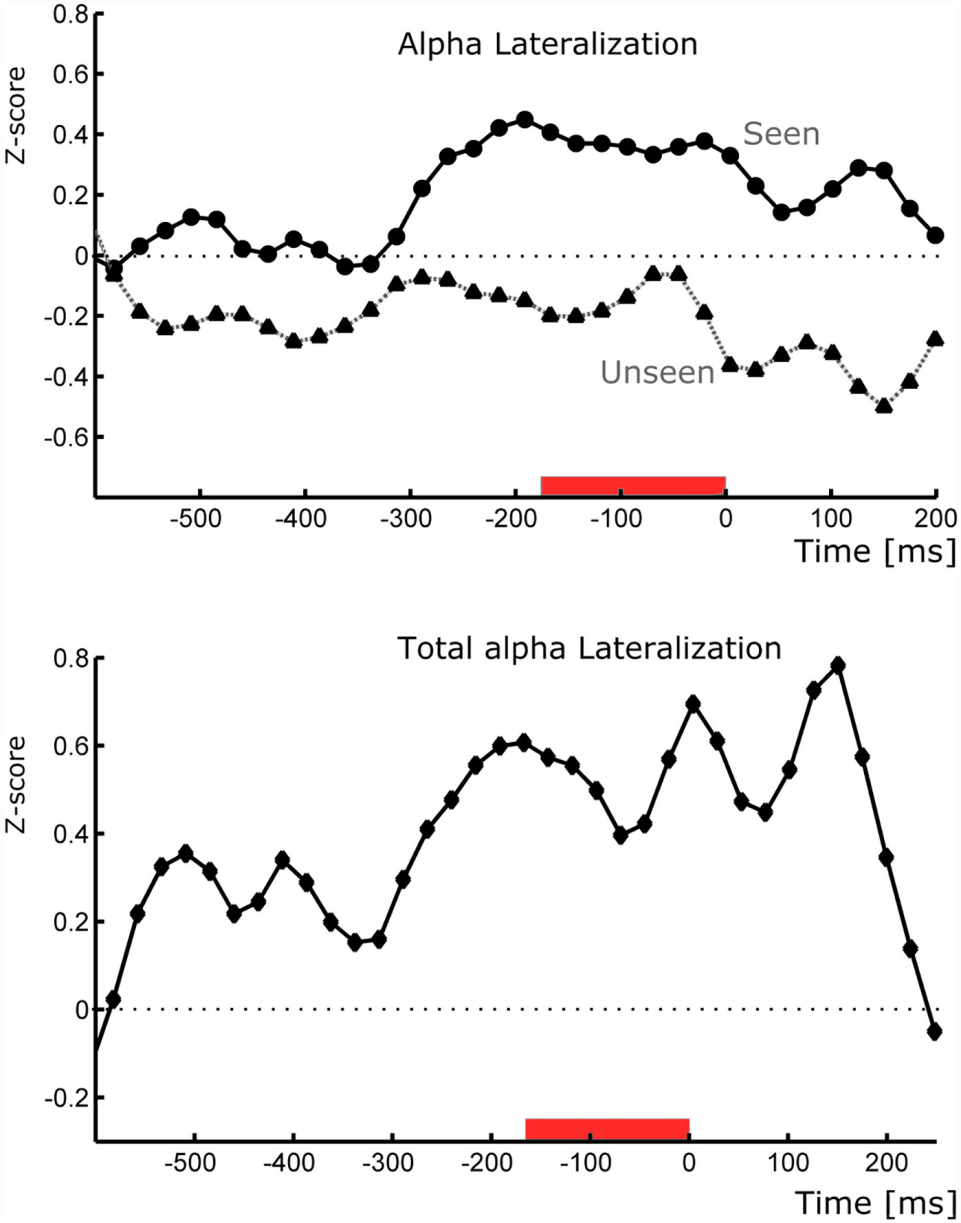
Time signature of alpha lateralization. **(Top)** Alpha lateralization, defined as the difference between ipsilateral and contralateral alpha power, as a function of time for Seen and Unseen conditions. **(Bottom)** Total lateralization defined as the difference in lateralization between Seen and Unseen conditions as a function of time. Time cero indicates the start of the target. Red bars indicate the time period where statistical significance was tested (p<0.05, Wilcoxon signed rank test).

**Fig 5:**
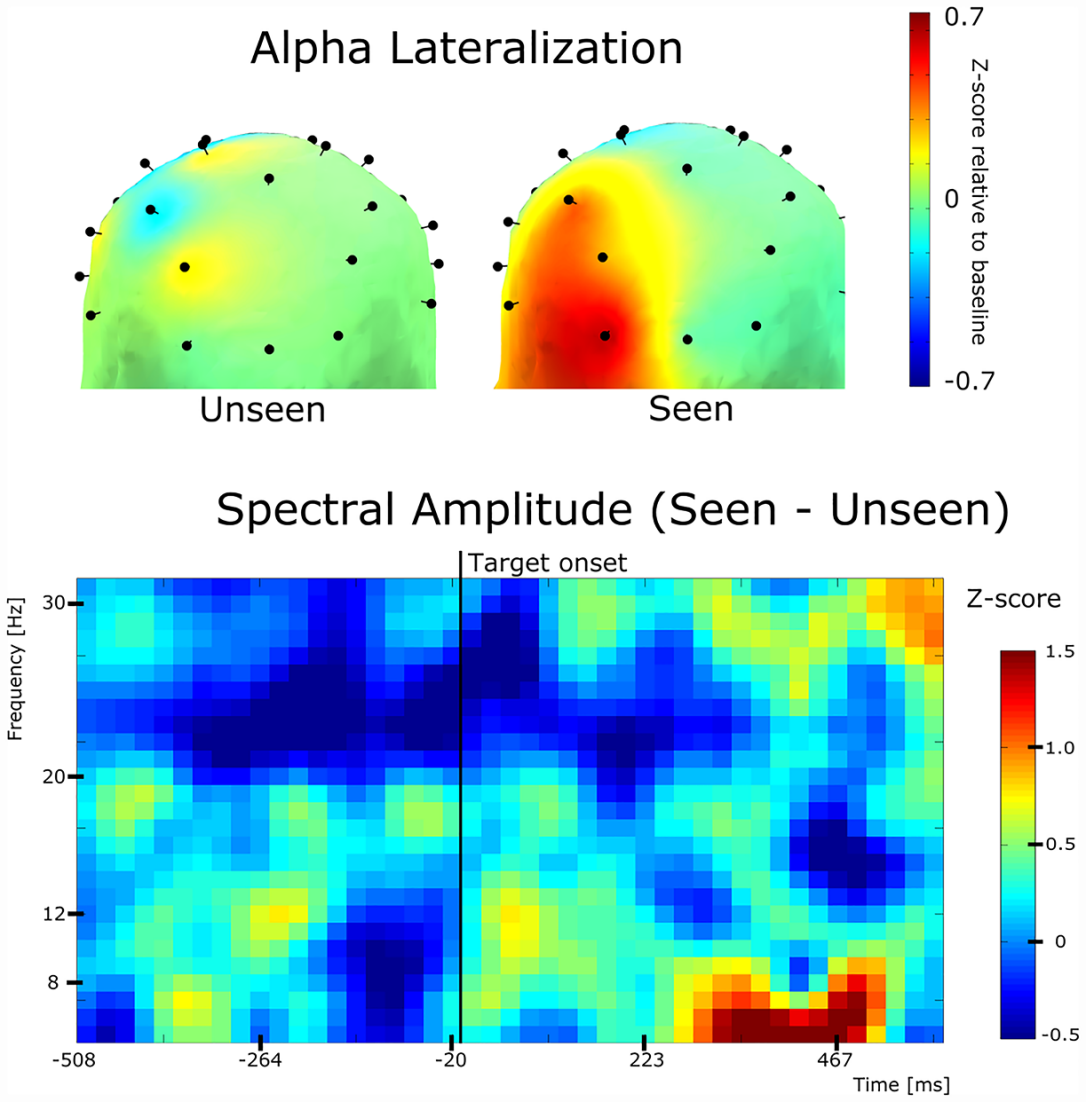
Lateralization distribution and general spectral differences. **(Top)** Half-scalp topoplots depicting the magnitude of lateralization for Seen and Unseen conditions. All of the data is presented on the left side of the scalp images (chosen arbitrarily, see Methods). It shows the topological distribution of alpha lateralization across parieto-occipital locations. **(Bottom)** Time-frequency plot of the differences in spectral amplitudes between Seen and Unseen conditions. Time cero corresponds to the beginning of target presentation.

We calculated alpha-power between 8–12Hz and in broad parieto-occipital regions of interest (ROI): accordingly to the 10–20 placement system [23] left ROI comprised electrodes O1, PO3, P3, P7, CP5, C3 and T7 while right ROI was composed of electrodes O2, PO4, P4, P8, C4, CP6 and T8. For the total alpha power we averaged across both ROIs for Seen and Unseen trials (Figs 3A, 4 and 5, bottom). We first computed the ipsilateral and contralateral alpha power for Seen and Unseen trials. To do this we averaged alpha power over scalp ROIs of the same side to where the target was presented (Ipsilateral) and the opposite side (Contralateral). This resulted in four measurements of alpha power, Seen Ipsilateral, Seen Contralateral, Unseen Ipsilateral and Unseen Contralateral (Fig 3B). To isolate the lateralization effect we calculated the Ipsilateral minus the Contralateral alpha power for Seen and Unseen trials (Fig 3C; see also Thut et al., 2006). Alpha power was calculated for a fixed time window immediately before target onset (-170ms to 0ms, Fig 3) for statistical analysis. We were also interested in exploring the time signature of this phenomenon. To do this we analyzed the lateralization for Seen and Unseen trials separately (Fig 4, top) but also their combined effect (Fig 4, bottom, Seen—Unseen).

To explore the general oscillatory brain activity evoked by our stimulation paradigm we constructed a time-frequency plot using the alpha power (z-score) of the combination of the previously defined ROIs (Fig 5, bottom).

### Half-scalp plots

Next we wanted to explore the scalp distribution of alpha-band lateralization. To do this we calculated the electrode-by-electrode alpha lateralization, in contrast to the broader effect (Figs 3 and 4). We first defined 14 analogous left-right electrode pairs (e.g. PO3, PO4). Lateralization was computed as the subtraction of the ipsilatateral electrode minus the contralateral electrode of each electrode pair. Thus 32-channel data was transformed into 14-channel data (14 analogous pairs, 4 central electrodes). We depicted this as half-scalp plots in which only 14 of the 32 points have relevant information (the rest of the scalp positions were assigned zero values. For visualization purposes we chose to present this information in a 3-D scalp plot with an occipital view, using only the left side (Fig 5, top).

All data is presented as mean ± standard error of the mean (SEM) unless otherwise stated. Statistical analysis for pairwise comparisons was done with a non-parametric paired test (Wilcoxon rank sum test) with a threshold value for alpha of 0.05 unless otherwise stated.

## Results

### Behavioral data

The probability of target detection and the number of distractors per frame during the calibration phase robustly co-varied (Fig 2, top). This was evidenced by a significant and strong correlation between these parameters at the subject average level (Spearman correlation; p < 10^-7; r^2 = 0.95). The mean detection rate in the post-calibration phase was 54.3% (SEM = 7.0%). This detection rate was consistent across time (Fig 2, bottom). However, we found a significant negative correlation between the detection rate and the ordinal block number during the post-calibration phase across subjects (Spearman correlation; p = 0.0041, r^2 = 0.15. The small magnitude of this effect is consistent with the constant number of distractors presented during this period. This minor negative correlation could be explained by the progressive fatigue of subjects. All of the above supports the effectiveness of our calibration phase in producing a stable detection rate of approximately 50%.

### Alpha-Power

Previous works have reported that alpha power across parieto-occipital scalp electrodes predicts subject’s behavior in some tasks [5–9]. Accordingly, we expected a lower total alpha power preceding the detection of the target, and a higher alpha power before unseen targets. To test this we computed the total alpha power for seen and unseen trials during a time window immediately preceding target presentation. Importantly, we found no difference in the total amount of alpha power for Seen and Unseen trials (Fig 3A, p>0.05). Next we explored whether alpha power before target presentation could differ between ipsilateral and contralateral scalp locations relative to the side of presentation of the target, as suggested by previous literature using informative cues. Fig 3B depicts the total alpha power of the four resulting conditions. None of the apparent differences between these conditions reached statistical significance. However to better explore this effect we analyzed the lateralization effect by subtracting the z-score of alpha power of Ipsilateral and Contralateral for both conditions. Fig 3C shows the significant difference between the lateralization effects of Seen trials versus Unseen trials. This lateralization effect was also significantly greater than zero for Seen trials (p<0.05). This showed no more than a tendency for unseen trials. This shows that alpha power is significantly lateralized towards the ipsilateral side, in detriment of contralateral, preceding correct target detection in the absence of informative cues.

In light of these results we were interested in exploring the time and scalp distribution of this spontaneous alpha-band lateralization. To do this we analyzed the dynamics of alpha lateralization in several time periods preceding target presentation. We analyzed both the separate lateralization effects of Seen and Unseen conditions (Fig 4, top) and the conjunct effect (Fig 4, Bottom). A consistent increase in the total alpha lateralization can be seen in the ≈600ms preceding the target. This effect was corroborated by the significant and positive correlation found between the total lateralization and the block's ordinal number (t = -600ms to 0; Spearman correlation; p<10^-3, r^2 = 0.84). We used block’s ordinal number instead block’s mean time because variable resting periods made block’s starting time mildly uneven across subjects. This result evidences an increasing lateralization of alpha power before the presentation of the target over the previously defined parieto-occipital ROIs. In order to characterize the scalp distribution of this alpha-band lateralization effect we constructed half-scalp topological plots (see Methods). Lateralization was calculated as the difference between ipsilateral and contralateral alpha-power (Z-score) of analogous electrodes on the left and right of the scalp, e.g O1 and O2. Alpha lateralization magnitude is depicted only in the left side of the half-scalp topoplots (right side is uninformative, see Methods). This is not to be mistaken as to reflect that only the left scalp side showed alpha lateralization. Fig 5 (top) shows the marked difference in lateralization magnitude between Seen and Unseen conditions.

Finally, as a way of exploring the overall spectral differences between Seen and Unseen conditions, we constructed a grand-average time-frequency chart (Fig 5, bottom). It ilustrates the lack of significant differences between conditions in the ≈200ms preceding target presentation in the alpha range (see Fig 3).

## Discussion

Attention is a selective process, allowing for the differential processing of the elements that we encounter. One approach in which this has been studied and modeled is by using the concept of saliency [24]. Under this model, feature-maps are extracted from the bottom-up information of the visual scene [25] and combined into a coherent saliency map [26,27]. This map marks the differential importance or behavioral relevance of elements on the visual scene, thus guiding more detailed inspection or directly promoting a particular behavior. This model fits very well with classical cue-stimulus visuospatial attention paradigms [4,28]: a first stimulus, for example an arrow, points towards one side of the visual scene indicating where a future target stimulus will appear, thus guiding attention. An interpretation of this paradigm is that the cue modifies the corresponding saliency map, thus prompting an enhanced response towards one particular side. Electrophysiological evidence of such visuospatial attentional modulations have been extensively shown in the form of differential alpha-power lateralization in response to informative cues (e.g. Thut et al., 2006). Here we show the same electrophysiological signature in the absence of bottom-up information that could modify the respective saliency map; the visual scene preceding target was dynamical but homogeneous. This contrasts with research on alpha dynamics for example in the exploration of natural scenes where in the same way no explicit cue is given. However the complexity of natural visual scenes does generate a rather rich saliency map, which in turn guides attention based on visual information. We interpret our results as evidence for an unguided or spontaneous component of attention dynamically functioning with independence of directing bottom-up information. Models of visuospatial attention, in particular of the functioning and construction of the saliency map, indeed include non-deterministic components [17]. They emphasize that, for example, stochastic fluctuations of the saliency of particular objects or features, aid in the functional modelling of the visual system [19,29].

Interestingly, we did not observe differences in the total alpha power between Seen and Unseen conditions in parieto-occipital sites in the ≈200ms preceding target presentation, as reported in previous works using informative spatial cues (e.g. van Dijk et al., 2008). This suggests that the subjects' general alertness did not differ between Seen and Unseen trials. Here we only observed differences in the topological distribution of alpha-band activity. Contributions for this lateralization effect appear to come differentially from ipsilateral and contralateral scalp locations. Although not statistically significant, Fig 3B suggests that lateralization was particularly driven by alpha power increases rather than decreases. Both Seen and Unseen conditions show an increase in alpha power before target presentation respect to the baseline period. Apparently this increase in alpha power, when at the ipsilateral side of an upcoming target, increased the chances of subjects detecting it (Seen trials). Accordingly, when this increase occurred at the contralateral side respect to the target presentation, subjects would be more likely to overlook the target (Unseen trials). This contrasts with work showing decreases of alpha activity over contralateral scalp sides rather than increases over ipsilateral areas for period preceding correct target detection (e.g. Cosmelli et al., 2011). These putatively stochastic alpha-band increases could be the reflection of subjects guessing the side of the future target. However this was not the instruction and no subject reported having systematically done this during the experiment. Nevertheless further scrutiny of this phenomenon is required to elucidate whether voluntary guessing or automatic attentional fluctuations account in a better way for this phenomenon. We also believe that a more refined study of the time-dynamics on different scalp localization of alpha-band lateralization would be of great importance.

Here we report significant parieto-occipital alpha lateralization previous to target stimulus presentation in the absence of previous bottom-up information about the upcoming target. The present work serves as evidence of an unguided or spontaneous component of attention, thus contributing in two main ways to our understanding of attention. First it advances un-cued signal detection paradigms as a viable experimental strategy to study this spontaneous attention component. Analysis of alpha-band lateralization in signal-detection paradigms using masking or degraded stimuli to difficult target detection could be of great significance. We believe that the lack of a cue previous to target stimuli allows a better observation of spontaneous brain dynamics and the related attentional processes. On the other hand, because no preceding information about target location was delivered, we hypothesize that alpha-power lateralization is likely to occurs continuously and spontaneously under this experimental paradigm. This is especially noteworthy as we show that spontaneous ongoing dynamics of alpha power can appear as strong modulators of behavior. This evidences that, for example, in the analysis of neural responses evoked by a particular stimulus, the neural responses obtained are a combination of the effect of the stimulus but also of the ongoing brain activity. Thus, it becomes relevant to better characterize these spontaneous dynamics, in particular of alpha-band topography. In conclusion we believe that the present results are of importance to dissociate spontaneous from guided attentional processes.
